# Progressive Cavity Pumps—A Comparison of New Technology for Gradient Bioprinting to Existing Extrusion Methods

**DOI:** 10.1002/elsc.70070

**Published:** 2026-02-23

**Authors:** Franz Moser, Shiva Rahmani, Tomasz Jungst

**Affiliations:** ^1^ Department for Functional Materials in Medicine and Dentistry Institute of Functional Materials and Biofabrication University of Würzburg and Key Lab Polymers for Medicine of the Bavarian Polymer Institute (BPI) Würzburg Germany

## Abstract

Extrusion‐based Bioprinting is a key technology in biofabrication, yet the choice of extrusion method is often limited to established techniques built into most bioprinters, limiting the print fidelity and more demanding applications like printing material gradients. In this technical report we compare the emerging method of progressive cavity pump with established technologies such as pneumatic extrusion and syringe pump‐based printing setups. The three methods were compared for their accuracy and precision in extruding 35% Pluronic F127, followed by test simulating different flow profiles, material dependency and ability to transfer between hardware setups. The progressive cavity pumps showed the most advantageous behavior for gradient printing, with the syringe pumps needing more iterations for stable extrusion and the pneumatic extrusion enabling high‐volume extrusion but showing lower precision. This was further shown with the printing of different gradients.

## 1 Introduction

Biofabrication is an emerging research field that combines principles from biology, medicine, engineering, and material science to fabricate functional tissues to bridge the gap between 2D culture and in vivo models. Using additive fabrication methods, specifically tailored materials are processed into complex three‐dimensional constructs [1]. These materials are so‐called bioinks consisting of cells and hydrogels as supporting material to give the constructs tissue‐like mechanical properties and desired abilities but also provide the cells with adhesion motifs and an effective nutrient supply. To achieve this, the materials have a high water content and a low polymer content for ideal mechanical properties but still enable maximum nutrient diffusion [2].

The aim of biofabrication in this case is to create tissue models to improve disease models and enhance pharmacological testing to close the gap between 2D cell culture and animal testing. To achieve biological tissue functionality, a wide range of parameters coming from the three pillars of material properties, cells, and biochemical cues must be met. From a fabrication point of view, one of the most challenging parts of this is the recreation of naturally occurring tissue architecture. As most natural tissues have no constant material properties within their hierarchical organization, this means either changing printing parameters drastically during printing or using multiple materials [3]. The hierarchical organization of tissues is usually changing gradually, which is why the printing of gradients is of high interest to the field.

A gradient is a continuous change of one or more parameters across a space. Biological gradients therefore have a multitude of different occurrences; they can appear in a structural way due to density, as in bones, having a dense outer structure and a porous inner structure [4]. They can be in the number and type of cells as in cartilage with more stem cell‐like cells in the deeper levels and differentiated chondrocytes on the surface, or a gradient of different signaling factors or physical cues to increase angiogenesis toward tissues with lower oxygen concentration, to give a few examples [5].

Recreating these gradients has been the focus of several studies in the recent past, but it remains a challenge in biofabrication. Traditional 3D printing methods are not directly transferable and often not applicable to bioinks, as temperatures or shear stresses are not compatible with the integrated cells or the used hydrogel materials. Gradient printing in biofabrication has been shown with pneumatic extrusion methods, though either using complex multimaterial setups [6] or special flow effects in cartridges with no in‐process control [7]. Systems using syringe pumps (SP) were able to create very complex and detailed gradients of different material properties [8]. However, the gradients were created using very low viscous materials in highly specialized setups in combination with other technologies, limiting the use of inks to focus, for example, on shape fidelity [8, 9]. More recently progressive cavity pumps have also been used for gradient printing, showing highly controlled gradients, but also using specialized setups [10]. Progressive cavity pumps operate using a rotating helical rotor within a stator, forming sealed cavities that transport the material axially with each rotation. The resulting flow rate is proportional to rotor speed and largely independent of material rheology. Backpressure is only needed for feeding material into the mechanism. This enables stable, continuous extrusion of various materials with a high degree of control. These methods represent the most prevalently used strategies in extrusion bioprinting [11].

This work aims to compare three extrusion methods used in bioprinting (pneumatic, syringe, and progressive cavity pumps), examining their accuracy and precision in dispensing standard hydrogel inks (Pluronic and Alginate) at typical flow rates. By benchmarking their susceptibility to changes in flow profile, material, or system parameters, we show their usability for gradient printing of two materials. This study analyzes whether the use of progressive cavity pumps is superior for bioprinting with a focus on gradient printing.

## 2 Materials and Methods

### 2.1 Materials

#### 2.1.1 Pluronic

Pluronic F127 (Merck, Germany) was added to ddH_2_O at 35 wt%. The mixture was shaken and stored at 8°C overnight to ensure complete dissolution. Before printing, it was filled in either a cartridge or syringe in fluid form and heated to 37°C for 15 min while moving, ensuring a homogeneous solid state. The density of 35% Pluronic F127 was measured at 1.05 g/mL.

#### 2.1.2 Alginate

Alginate PH176 (Vivapharm, Germany) was added to 37°C ddH_2_O at 5 wt% while stirring. The mixture was kept overnight at 37°C and stirred again to ensure full dissolution. Before printing, it was transferred into cartridges using a direct displacement pipette. The density of 5% alginate was measured at 1.05 g/mL.

## 3 Methods

### 3.1 Pneumatic Pump (PP)

The PP setup was built using the pressure control in a Bioscaffolder BS5.3 (Gesim, Germany). The material‐laden cartridge with a piston (Nordson, USA) was connected to the system's pressure tubing and controlled using the proprietary software.

### 3.2 SP

The SP system used was an AL‐1000 pump (WPI, United Kingdom). The pump was set up with a water‐filled 5 mL syringe and connected via water‐filled non‐elastic tubing without air bubbles to a cartridge containing the hydrogel material, ensuring the conservation of the solutions by a watertight piston in the cartridge while extruding the material hydraulically. Unless stated otherwise, a pause of 2 min followed each extrusion to allow a pressure equilibrium.

### 3.3 Progressive Cavity Pump

The progressive cavity pumps (PureDyne, Germany) were fitted to a modified 3D printer. The hydrogel material was loaded into the fitting cartridge, and the cartridge was loaded into the pump setup with the rotor in place. Volume and velocity of the extrusion flow were controlled via G‐code.

### 3.4 Different Extrusion Programs

The PPs were controlled via the Gesim software for specific pressures (deviation of 1 kPa) for a set time, the SPs were programmed for a given flow rate and volume, and the Puredyne pumps were controlled via G‐code within the printer control.

The extrusion program used for analyzing the initial extrusion and the influence of material and nozzle size for all pumping systems was set at a flow rate of 200 µL/min for 30 s to extrude a total volume of 100 µL.

The break profile was programmed for two 15 s extrusion phases at 200 µL/min with a 10 s break in between. The increasing flow rate on the SPs was set up by having three phases at 150, 200, and 250 µL/min for 10 s each. The PP was set up similarly, but with the extruding parameter being pressures of 57, 60, and 62 kPa for 10 s each, calibrated to the needed flow rates. The progressive cavity pump setup was programmed for flow rate and volume, so they were programmed to the same flow rates of 150, 200, and 250 µL/min and the corresponding volumes of 25, 33,3, and 41,7 µL, respectively. The decreasing flow profiles were programmed with the same parameters in reverse order for all extrusion systems.

The tuned printing for the SPs was done by increasing the flow rate to 128% (256 µL/min) to counteract the under‐extrusion of 35% Pluronic F127.

The used nozzles were G22, G23, and G27 (Nordson, USA), and extrusion was measured by weighing the extruded material.

### 3.5 Printing of Gradient Structures

The gradient extrusion was managed by using dual SPs or progressive cavity pumps. The two material‐filled cartridges were connected to a 2K mixer unit (C.Hafner GmbH, Germany), with the dead volume of the mixer accounted for. The mixer and the cartridges were mounted with custom holders for each extrusion method to a specially modified 3D printer (based on a Geetech A20M with a Duet 2 WiFi V1.04 mainboard). For the gradient printing, the ratio of extrusion of the two materials was changed by changing the volume and flow rate to still result in a total flow of 200 µL/min but change the material gradually but completely within the 10 steps while printing the 2D and 3D gradient structures. For the gradient printing with different materials, the mixing unit was exchanged for a G22 nozzle (Nordson, USA).

## 4 Results and Discussion

### 4.1 Accuracy and Precision

**FIGURE 1:**
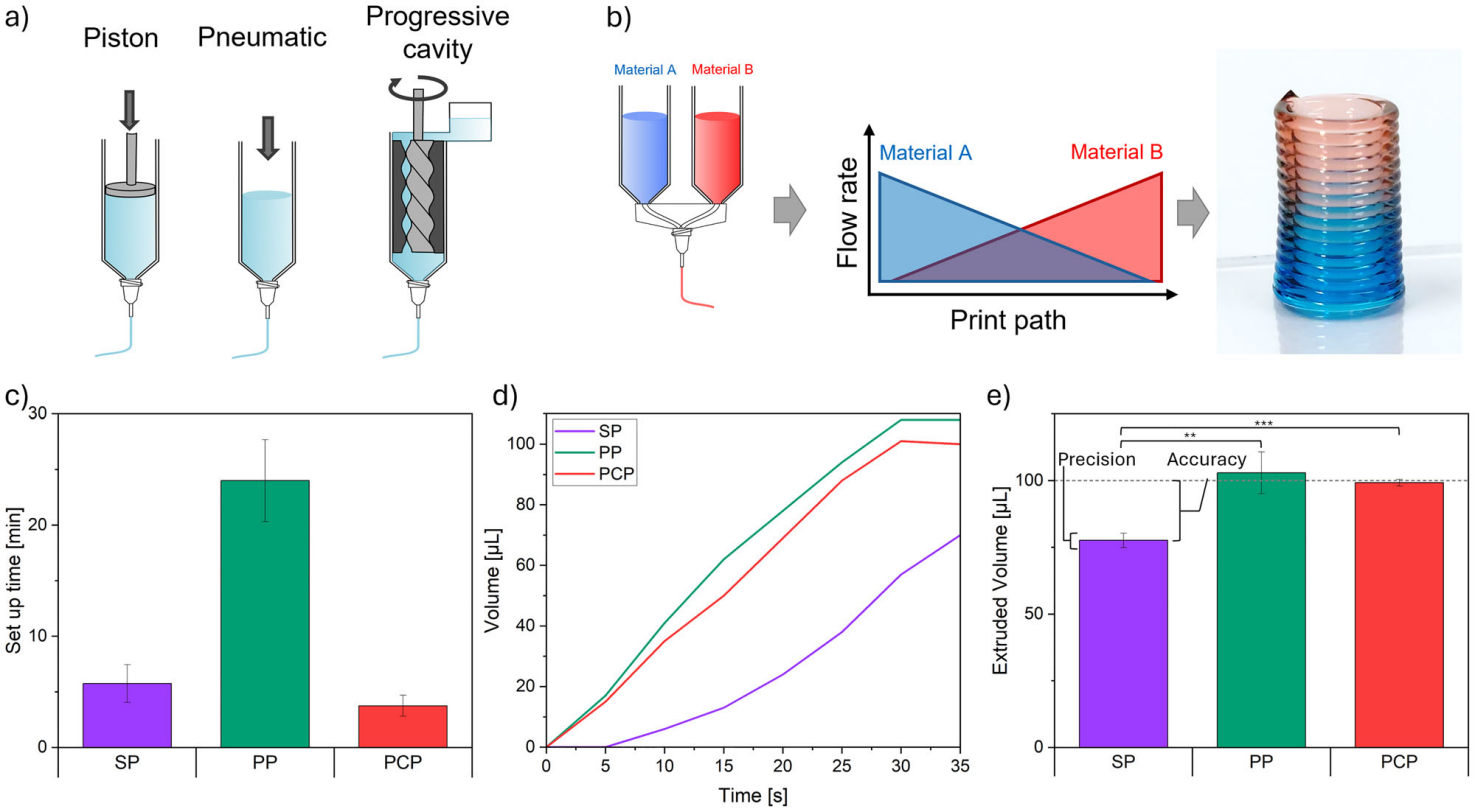
Schematic setup of syringe pumps with piston, pneumatic pumps, and progressive cavity pump (a). Schematic of gradient printing setup with two different materials, change in printing profile for gradient printing, and printed three‐dimensional gradient construct (b). Time needed for setup (c), material flow in 30 s extrusion (d), and amount of extruded material in 30 s (e) for SP (violet), PP (green), and PCP (red). The extruded material is Pluronic F127. Error bars indicate the standard deviation (SD; *n* = 4). *p < 0.1, **p < 0.05, ***p < 0.01 by one‐way ANOVA with Turkey's post hoc test.

The extrusion methods compared for their accuracy were a pneumatic system built into a 3D bioprinter (PP), a SP, and a progressive cavity pump (PCP) depicted in Figure 1a–c. All pumps were set up to extrude 100 µL in 30 s, resulting in a flow rate of 200 µL/min, comparable to commonly used flow rates for bioprinting [12].

The time needed for setup varied highly between the extrusion systems. The SP with 5.8 ± 1.7 min and the PCP with 3.8 ± 0.9 min could be programmed to extrude defined volumes, while the PP had to undergo iterations of fine‐tuning the pressure to extrude the desired flow rate, resulting in 24.0 ± 3.7 min time to set up (Figure 1e).

The difference in setup between SP and PCP is due to the SP needing to have contact of all moving parts and building up pressure in the entire system for ideal force transmission; this causes the first extrusion after inserting the syringe in the pump to not extrude the right amount. These pressure build‐up effects are also visible when comparing the amount of material extruded over the 30s (Figure 1f). While PP and PCP have a nearly linear increase in extruded material, the SP showed a delayed and non‐linear extrusion. These effects have been described before and are caused by compressibility in the system and the biomaterial [9].

The SP setup also showed a delayed start and end of extrusion compared to the programmed 30 s and even further after the depicted 35 s when not using the hydraulic force transfer and extruding directly from the syringe, with both having extruded close to 100 µL after 60 s. The setup with hydraulic force transfer was used and is shown since it is more easily incorporated into existing bioprinting setups than integrating two syringe pumps directly into the moving printhead. The PP shows greater deviation from linear extrusion than the PCP while also extruding more material in total. This is due to the intrinsic pressure regulation having a deviation of around 1 kPa. PCPs utilize a rotor's motion for continuous volumetric displacement of defined cavities of material and air pressure only for feeding material into the mechanism; therefore, they don't suffer from the pressure‐dependent or ‐buildup effects as SPs and PPs [13].

The relationship between pressure (force) and extruded material (volume) in shear‐thinning materials like most hydrogels is not linear, which, taken with the pressure variation, results in less constant and more deviating extrusion [14]. This is also visible when comparing the extruded material over different runs (Figure 1g). Accuracy is describing the deviation of the mean to the programmed value, and precision is describing the standard deviation (Figure 1g) [13]. While both PP (102.95 ± 7.80 µL) and PCP (99.25 ± 1.30 µL) showed high accuracy, the PP had the least precision of all three methods. SP showed high precision but lower accuracy with 77.60 ± 2.73 µL. Only the PCP showed both accuracy and precision, validating previous studies [13]. Combined with the linear extrusion and quick setup time, they showed high usability for the exact extrusion needed for gradient printing (Figure 1d).

It should be noted that the accuracy of the SP setup increased when extruding multiple runs one after another, with only short pauses in between. This is, however, not practical for the intended purpose of gradient printing, as the extrusion also continued after the programmed 30 s due to the pressure buildup. Therefore, it could not be integrated into the method.

### 4.2 Influence of Changes in Flow Profile

**FIGURE 2:**
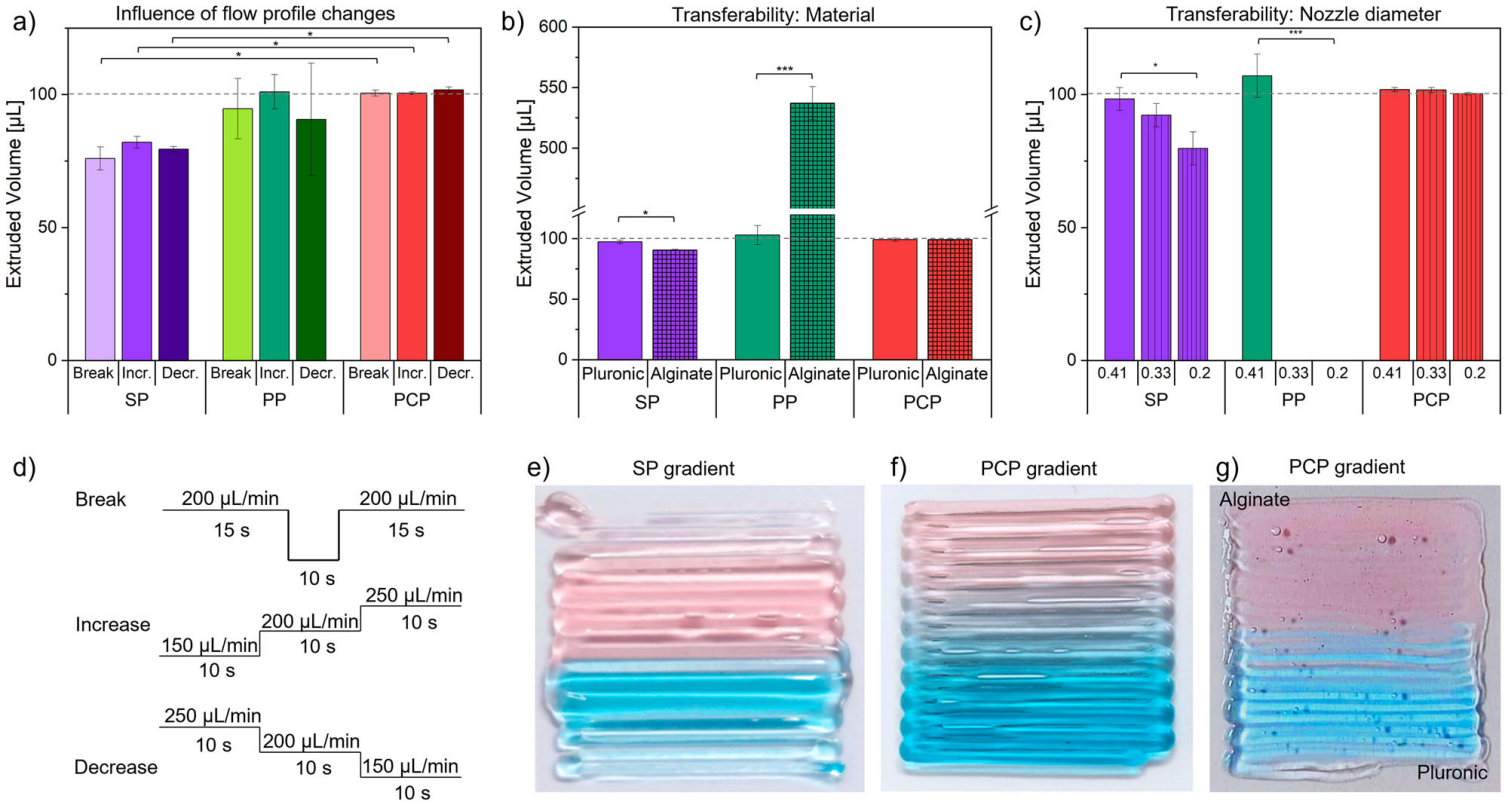
Extruded volume of Pluronic F127 for SP (violet), PP (green), and PCP (red) when varying extrusion profiles (break [lighter shade], increase [standard], decrease [darker shade]) (a), extruded material (35% Pluronic [standard], 5% alginate [plaided]) (b) and nozzle diameter (0.41 mm [standard], 0.33 mm [stripes light], 0.21 mm [stripes intense]) (c). Printing profile of break, increase, and decrease shown in detail (d). Printed gradient of SP with two Pluronic inks (e), PCP with two Pluronic inks (f), and alginate (red) and Pluronic (blue) inks (g). Error bars indicate the standard deviation (SD; *n* = 4). *p < 0.1, **p < 0.05, ***p < 0.01 by one‐way ANOVA with Turkey's post hoc test.

The extrusion behavior was further investigated by how differently programmed flow profiles influence the extruded volume. Every printing path pauses in flow, increases in flow rate, and decreases in flow rate. All three extrusion setups were therefore examined for the impacts of flow breaks and increasing and decreasing flow rates (Figure 2a). The total programmed volume was 100 µL in all profiles. The included break was 10 s with flow rates of 200 µL/min. The increasing profile was in 10 s increments of 150, 200, and 250 µL/min and reversed for the decreasing profile, both without breaks (Figure 2d).

The relationship of accuracy and precision remained comparable to the constant extrusion. PP and PCP showed higher accuracy, while SP and PCP showed higher precision, with PCP exceeding the others in both categories, SP even significantly. None of the flow profile changes led to significant differences within each pumping system. However, in SP and PP, the increasing flow rate showed slightly higher extrusion values within their groups. This could stem from the fact that with increasing extrusion amount, the corresponding rise in pressure gives the system less room to adjust. As a result, there is less delayed extrusion. The PCP showed slightly higher values in extrusion amount when decreasing the flow rate compared to break and increase, though these were not significant.

In comparison, the PCP setup showed the highest stability across flow changes, which is necessary in accurate bioprinting, especially when printing gradient structures. Gradient structures also are printed by precisely controlling the ratio of two or more materials, which results in a more complex machine setup; therefore, a high degree of control is required [11].

### 4.3 Transferability

The influence of programmed flow profiles has been shown for the different extrusion setups. To further test the capability for gradient printing of the setups, it is important to compare the transferability between different materials, as printed gradients require two or more materials with different flow characteristics. To simulate this, two different hydrogel compositions, 35% Pluronic and 5% alginate as common in bioprinting, were loaded into the setups [13]. For comparability, the programming of the SP was adjusted to offset the under‐extrusion by increasing the flow rate to 256 µL/min. This ensured the extrusion of 100 µL of 35% Pluronic in 30 s. The programming of all setups for the extrusion of 100 µL in 30 s was kept, but the extruded material was changed from 35% Pluronic to 5% alginate to compare the transferability of the parameters between the different materials. Pluronic was extruded to the programmed 100 µL with the already observed deviation (Figure 2b). The SP showed a small but significant drop in accuracy to 90.6 ± 0.6 µL in the extruded amount of alginate. The PP showed a significant increase when extruding alginate of 537.2 ± 13.7 µL. The PCP showed constant extrusion of 99.2 ± 0.7 µL (alginate) and 99.3 ± 1.3 µL (Pluronic). In the extrusion of alginate, the SPs were the most precise, with the PCP nearly equal and the PP the least precise. The loss in extrusion accuracy of the SP shows the material dependency despite the displacement‐based mechanism. The large deviation in the PPs is most likely caused by the difference in viscoelasticity of the two shear‐thinning materials. The applied force causes higher flow of the alginate, which flows at lower pressures and therefore shows greater extrusion volume than Pluronic at the same pressure [15]. The PCP displaced a fixed volume right before the nozzle, so only a small amount of material is affected by its flow properties—unlike the SP, where the entire loaded volume is influenced.

Transferability was further evaluated by changing the nozzle diameter, altering system resistance, and showing adaptability of the different pump systems to hardware changes. The SPs showed reduced extrusion volume by ∼20 µL/min for each reduction of nozzle diameter of about 0.1 mm with the same programming (Figure 2c). The PP showed no extrusion with smaller diameter nozzles. The PCP showed no significant difference in extrusion when comparing the three nozzles. The flow rate and the pressure needed to extrude through a given tube are dependent on the quadrupled radius, which explains the strong effect with PP and the dependency of the SPs in the same manner as the material dependency.

### 4.4 Gradient Printing

Both accurate and precise extrusion are a necessity for gradient bioprinting. Two (or more) materials, which may differ in rheological properties, need to be extruded at defined volumes and flow rates to be able to form a gradual change between them.

PP was not used for gradient printing in this study. While gradient printing using PP is possible [16], it requires extensive calibration and use of materials with very similar rheological properties under tightly controlled conditions. Since this study focuses on gradient printing across a broad range of different biomaterials with minimal effort and setup time, PPs were not used.

The presented gradient structures were printed from 35% Pluronic colored by red and blue food coloring to facilitate qualitative analysis of the printed gradients. While more specific quantification of gradient composition is possible, this was not the scope of this study, but others [3, 12, 17, 18] The SP setup printed the intended color gradient, though not gradually (Figure 2e). The flow rate was not constant, evident by both the beginning and end not being extruded properly while the middle of the construct was being over‐extruded. This is caused by the nonlinear pressure buildup in the syringes, leading to mismatched internal pressure and actual flow rates. While optimized solutions adapted to this problematic exist, this setup reflects commonly used systems not specifically optimized for gradient printing.

The PCPs showed constant extrusion along the way and also a visibly gradual change in color (Figure 2f). The high degree of control also enabled the printing of gradient structures out of materials of different behavior like alginate (red) and Pluronic (blue) (Figure 2g) or even three‐dimensional gradient constructs (Figure 1d). The gradient of alginate and Pluronic was printed without a mixer using just a nozzle, causing the materials to not be mixed. This was done as the different flow characteristics caused the materials to not pass the mixer at the same time, changing the extruded material composition from the programmed. Further research on designs of mixing devices. Both of which were not feasible with the other methods and the same amount of preparation.

## 5 Conclusion

This study compared three common extrusion systems—pneumatic, syringe, and progressive cavity pumps—regarding their accuracy, precision, and adaptability in bioprinting scenarios and established a simple evaluation protocol to evaluate the feasibility of extrusion‐based systems for the generation of gradients. The progressive cavity pump (PCP) consistently demonstrated both high accuracy and high precision, independent of changes in flow rate, material composition, or nozzle geometry. The syringe pump (SP) showed high precision but struggled with under‐extrusion and significant sensitivity to material and hardware changes, limiting its reliability for gradient applications. The PP, while accurate in some cases, exhibited poor precision and was highly dependent on both material properties and pressure control, making it less suitable for complex tasks like gradient printing. Both SPs and PPs can be optimized to extrude with the needed parameters, but this takes time and needs to be repeated for changes in material. Due to the continuous positive displacement of the individual hydrogel cavities, PCPs can be programmed with a high degree of control and little material dependency. While some of these shortcomings can be minimized with technical advances and specific adaptations, especially in SPs, the PCPs showed the highest control with little technical effort once the pumps were integrated into the system.

Taken together, these results validate the superior performance and robustness of PCPs for advanced bioprinting tasks, particularly when precision across dynamic conditions is essential. For bioprinting applications there are already some insights into the influence of PCPs on cell printing, but it needs to be analyzed further for broader integration of the capable method into the field [12, 13].

## Funding

This study was supported by the Deutsche Forschungsgemeinschaft (DFG, German Research Foundation, Project number 326998133) within the Collaborative Research Center TRR225 (subproject B09).

## Ethics Statement

This study did not involve human participants or animals therefore did not require ethical approval.

## Conflicts of Interest

The authors declare no conflicts of interest.

## Data Availability

The data that support the findings of this study are available from the corresponding author upon reasonable request.
